# Peripheral mechanisms of arthritic pain: A proposal to leverage large animals for *in vitro* studies

**DOI:** 10.1016/j.ynpai.2020.100051

**Published:** 2020-07-28

**Authors:** Sampurna Chakrabarti, Minji Ai, Frances M.D. Henson, Ewan St. John Smith

**Affiliations:** a Department of Neuroscience, Max-Delbrück-Centrum für Molekulare Medizin (MDC), Berlin, Germany; b Department of Pharmacology, University of Cambridge, UK; c Department of Veterinary Medicine, University of Cambridge, UK; d Animal Health Trust, Newmarket, Cambridge, UK

**Keywords:** Arthritis, Large animals, Sensory neurons, *In vitro*, Pain models

## Abstract

Pain arising from musculoskeletal disorders such as arthritis is one of the leading causes of disability. Whereas the past 20-years has seen an increase in targeted therapies for rheumatoid arthritis (RA), other arthritis conditions, especially osteoarthritis, remain poorly treated. Although modulation of central pain pathways occurs in chronic arthritis, multiple lines of evidence indicate that peripherally driven pain is important in arthritic pain. To understand the peripheral mechanisms of arthritic pain, various *in vitro* and *in vivo* models have been developed, largely in rodents. Although rodent models provide numerous advantages for studying arthritis pathogenesis and treatment, the anatomy and biomechanics of rodent joints differ considerably to those of humans. By contrast, the anatomy and biomechanics of joints in larger animals, such as dogs, show greater similarity to human joints and thus studying them can provide novel insight for arthritis research. The purpose of this article is firstly to review models of arthritis and behavioral outcomes commonly used in large animals. Secondly, we review the existing *in vitro* models and assays used to study arthritic pain, primarily in rodents, and discuss the potential for adopting these strategies, as well as likely limitations, in large animals. We believe that exploring peripheral mechanisms of arthritic pain *in vitro* in large animals has the potential to reduce the veterinary burden of arthritis in commonly afflicted species like dogs, as well as to improve translatability of pain research into the clinic.

## Highlights

• A large proportion of analgesics fail to translate from rodents to humans.
• Large animals share anatomical and behavioral similarities to humans.
• Studying large animals can provide novel insights for arthritic pain.
• *In vitro* techniques developed in rodents can be adopted in large animals.

## 1 Introduction: Brief overview of mechanisms driving arthritic nociception and pain

“Arthritis” is derived from the Greek words “arthros” meaning joint and “itis” meaning inflammation. One crucial feature that the etymology of arthritis excludes is the concept of nociception and pain, although arthritis is a broad term encompassing musculoskeletal disorders in which chronic pain is the leading cause of morbidity (Neogi, 2013). Indeed, arthritic pain has been recognized and managed globally since antiquity. Between 1000 and 300 BCE, both the Indian medico-religious text Atharvaveda and the Greek philosopher Hippocrates, described the etiology of arthritis as pain originating from joints and spreading to the rest of the body (Sharma and Arora, 1973, Short, 1974). Modern research attributes the pain experienced first at the site of the disease (e.g. joints), and subsequently at other parts of the body, to peripheral and central components of pain respectively. Furthermore, Roman Emperor Claudius’ physician Scribonius Largus (~40 CE) described a chronic polyarthritis, which he treated by administering a shock of static electricity to the patient’s feet using torpedo fish, presumably in an attempt to modulate neuronal activity and thus suppress nociceptive input and the sensation of pain (Kellaway, 1946). From this brief look into history, it is clear that pain management by targeting peripheral inputs has been acknowledged by the medical community since ancient times.

The current understanding of arthritic pain is that disease progression causes marked changes in the function of non-neuronal cells (e.g. synoviocytes and immune cells, such as macrophages), which results in inflammation of the joint environment, and aberrant communication between these non-neuronal cells and sensory neurons at the site of the disease causes pain. Although differences exist between arthritic conditions, i.e. osteoarthritis (OA) pain is considered to be more degenerative in nature, primarily affecting cartilage and bone (French et al., 2017), whereas rheumatoid arthritis (RA) is perceived as more inflammatory (Walsh and McWilliams, 2014), the important role of inflammation in OA pain is becoming increasingly clear (Goldring and Otero, 2011, Miller et al., 2019, Neogi et al., 2016).

Joint-innervating nerves, the cell bodies of which are located in the dorsal root ganglia (DRG), detect both innocuous and noxious stimuli, the latter occurring through a subset of sensory neurons called nociceptors that transmit nociceptive information using a variety of strategies (which can occur exclusive to each other or in combination). Firstly, inflammatory mediators can directly activate joint nociceptors to fire action potentials (AP), for example, protons present in the inflammatory milieu can activate a variety of receptors expressed by nociceptors (Pattison et al., 2019). Secondly, peripheral sensitization can occur, whereby the threshold required for AP generation is reduced, which can result from changes in the sensitivity and/or expression of ion channels either involved in transduction of noxious stimuli (Dubin et al., 2012, Lechner and Lewin, 2009, Vellani et al., 2001, Zhang et al., 2005), or in AP generation (Staunton et al., 2013). Thirdly, a further form of peripheral sensitization involves the inflammatory milieu unmasking previously ‘silent’ nociceptors (reviewed in (Schaible et al., 2002), with recent evidence identifying nerve growth factor (NGF) as being key to unmasking silent nociceptors to become mechanically sensitive and thus provide extra nociceptive input (Prato et al., 2017). From the periphery, APs from joint nociceptors are transmitted to the dorsal horn of the spinal cord where they synapse with the spinal interneurons and projection neurons, although the molecular detail of this connectivity is poorly understood compared to our growing understanding of the spinal circuitry involved in cutaneous sensory nerve function (Peirs et al., 2020). In chronic arthritis, there is tonic nociceptive input, which is enhanced by peripheral sensitization, and this barrage of information being received by the spinal cord can lead to central sensitization (hyperexcitability in the central nervous system, reviewed in (Harte et al., 2018, Wood et al., 2019, Woolf, 2011)); there is also evidence that this effect might be longer lasting when it involves deep tissue nociceptors (Wall and Woolf, 1984). For example, one study found that in a model of chronic OA, injection of NGF into the knee joint can increase extension-evoked firing of wide-dynamic range dorsal horn neurons (Sagar et al., 2015). The three major mechanisms of central sensitization are 1) glutamatergic neurotransmission mediated (summation of sub-threshold excitatory post-synaptic currents from acute pain leads to AP firing in higher order neurons), 2) loss of tonic inhibitory controls due to disinhibition of γ-amino butyric acid receptors (GABA) and glycinergic pathways and 3) glia-mediated (Basbaum et al., 2009, Old et al., 2015). The glia-mediated mechanisms rely on inflammatory mediators, for example, elevated levels of interleukin 1β (IL-1 β) have been detected in the cerebrospinal fluid of RA patients (Lampa et al., 2012). The cytokine fractalkine (shown to be upregulated in protein isolated from human OA synovium (Gowler et al., 2019)) might also play a role in central sensitization because its receptor CX3CR1 is upregulated in spinal microglia following neuropathic pain generation in rats (Lindia et al., 2005). Indeed, it has been shown that the microglial protease, cathepsin S exerts pro-nociceptive effects in the central nervous system (CNS) by cleaving fractalkine from neuronal membranes which can then activate CX3CR1 receptors (Clark et al., 2009). Furthermore, in a rat model of RA, both a cathepsin S inhibitor and a fractalkine neutralizing antibody normalized mechanical hypersensitivity (Clark et al., 2012).

Advances in neuroimaging have also revealed the brain networks involved in processing of arthritic pain. Specifically, OA patients show disruption of resting state default mode network and a decrease in grey matter volume in the thalamus, as well as increased activity of the periaqueductal gray region (PAG, part of the descending pain modulation system) (Gwilym et al., 2010, Gwilym et al., 2009). Importantly, imaging of the PAG, nucleus cuneiformis and rostral ventromedial medulla has provided evidence that OA patients with neuropathic pain (as opposed to nerve injury pain) have a poorer outcome post-arthroplasty, thus suggesting that neuroimaging could be a useful tool to stratify patients (Soni et al., 2019). Overall, these results demonstrate that both peripheral and central mechanisms are important in arthritic pain and the direct behavioral outcomes of these pain generating mechanisms for the individual in pain: allodynia (in which a previously non-painful, innocuous stimulus causes pain) and/or hyperalgesia (in which a noxious, painful stimulus is perceived to be more painful).

### 1.1 Relevance and scope of the review

The relative importance of peripheral vs central pain mechanisms is unknown in arthritis, however, several lines of evidence demonstrate that controlling peripheral mechanisms of nociception can provide pain relief: 1) local administration of analgesics relieves arthritic pain (Creamer et al., 1996, Uziel et al., 2003), 2) peripherally restricted anti-NGF antibody administration relieves OA pain (Schnitzer et al., 2019) and 3) total joint replacement can provide pain relief in OA and RA (Neogi, 2013, Wolfe and Zwillich, 1998). Given the importance of pain originating from the periphery in arthritis, it is useful to understand the underlying mechanisms of nociceptor activation and peripheral sensitization to identify drug targets and subsequently develop therapeutics. This has led to the establishment of multiple pre-clinical *in vivo* and *in vitro* inflammatory pain models to simulate human arthritic pain, each of which has its strengths and weaknesses. The three main strategies used for generating arthritic pain in animal models are: 1) altering the joint environment by administering irritants that lead to direct tissue damage or recruit the immune system to attack joints 2) trauma that leads to either acute or chronic development of joint pain (induced models) and 3) utilizing animals that naturally develop arthritis. Currently, these experimental models are largely conducted in rodents, due to them being amenable to genetic manipulation, having a short reproduction time and ease/cost of housing. These *in vivo* rodent models of arthritis and the behavioral outcomes measured in such models have been extensively reviewed (Gregory et al., 2013, Krock et al., 2018, Kuyinu et al., 2016, Samvelyan et al., 2020) and hence this review will focus on *in vivo* models of arthritis in large animals. However, a review of *in vitro* models and assays for dissecting arthritic pain in the periphery is lacking, a gap this review will address in rodents and in large animals, and conclude that leveraging large animals for *in vitro* studies could potentially accelerate the field of arthritic pain research.

## 2 Potential for use of large animals in arthritic pain research

**Fig. 1:**
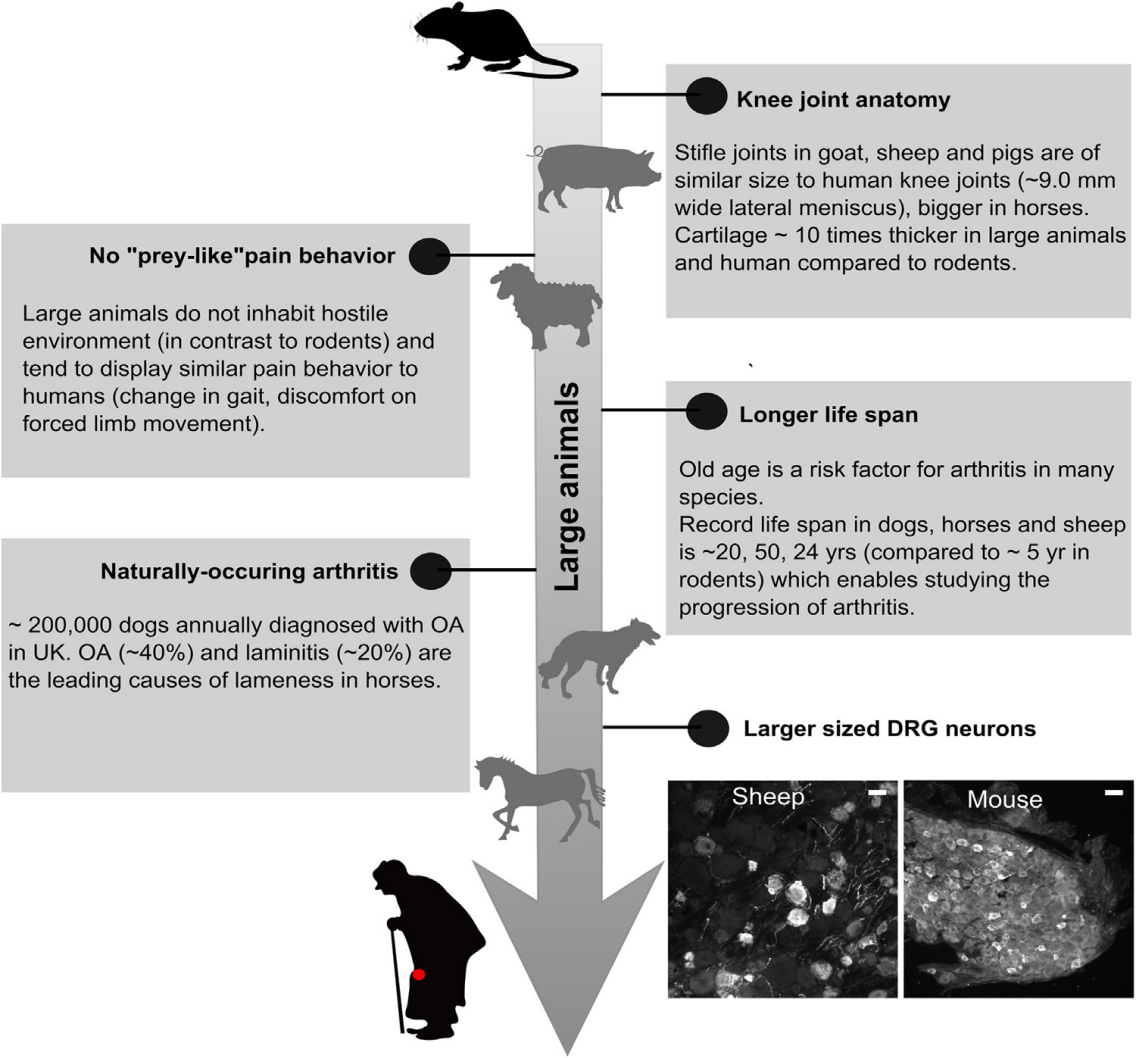
Schematic diagram emphasizing the potential for large animals in translational arthritic pain research. Large animals have similar sized knee and cartilage thickness compared to humans (McCoy, 2015, Proffen et al., 2012), longer lifespan (Carey and Judge, 2000), and larger DRG neurons compared to rodents (brighter neurons indicate CGRP immunoreactivity, Scale bar = 50 um). Unlike rodents which are prey species (Rice et al., 2008), large animals are less likely to hide pain behavior and are susceptible to naturally-occurring arthritis (mostly OA) similar to humans (K. L. Anderson et al., 2018; Centers for Disease Control and Prevention, 2015, Slater, 2016).

The inefficiency of translating therapeutics to humans following demonstration of efficacy in rodents has been a major concern for the pain community with a ~10% likelihood of FDA approval for studies entering a Phase I clinical trial (Hay et al., 2014). A number of reasons have been suggested for this translational gap including innate differences in rodent and human pain biology due to their phylogenetic distance (Blackburn-Munro, 2004, Klinck et al., 2017, Mao, 2012). In the context of preclinical research, large animals are considered to be animals larger than rabbits and rodents, for example horses, cattle, sheep, goats, pigs and dogs. Studying pain pathologies in these larger animals that are phylogenetically closer to humans, could potentially help bolster the translational potential of therapeutics, since these animals might share a greater sequence homology with the molecular drug target in man (Kruger and Light, 2010). For example, the pain managing drug for migraine, the calcitonin gene-related peptide (CGRP) receptor antagonist, MK-0974, was found to be > 10 fold more potent in human and rhesus/marmoset monkeys than in rodents because of greater sequence homology in receptor activity modifying protein 1 (RAMP1), which combines with the calcitonin receptor-like receptor to act as a receptor for CGRP (Hershey et al., 2005, Salvatore et al., 2008). The smaller body sizes and differences in drug metabolizing pathways of rodents compared to humans also complicate prediction of pharmacokinetics and drug efficacy. For example, pregabalin appears to be more rapidly effective in rodents than in humans, possibly due to smaller body size (Arezzo et al., 2008, Field et al., 1999), and, when considering opioid pharmacokinetics, cytochrome P450 2D (CYP2D), a key enzyme in the opioid metabolism pathway, has nine active forms in mice compared to one in humans (Dagostino et al., 2018, Ingelman-Sundberg, 2005). Rodents also tend to display less nocifensive/pain behavior than non-prey species since overt portrayal of pain behavior can hinder survival in nature, thus posing another barrier to translation (Rice et al., 2008). By contrast, dogs and horses typically live in less hostile environments and show similar pain behaviors to humans, which can be assessed (e.g. lameness grading) and validated (e.g. medical imaging techniques) using clinical procedures developed for humans, as well as being treated using anti-inflammatory and analgesic drugs in clinical practice (Meeson et al., 2019). Additionally, using large animals as model organisms provides specific advantages in the field of arthritis (summarized in Fig. 1). For example, large animals in general replicate human joint biomechanics better than rodents because of more similar joint anatomy to that of humans (Malfait et al., 2013, Proffen et al., 2012). In particular, cartilage and subchondral bone thickness in the joint of large animals, particularly in the horse, is more similar to humans than in small animals (average cartilage thickness in mouse = ~0.03 mm vs. horse = ~1.5 mm vs. human = ~2.0 mm) (Cook et al., 2014, Malda et al., 2012, McCoy, 2015).

Along similar lines, the diameter of DRG neurons is also greater in large animals, such as in sheep (unpublished observation), and humans (Rostock et al., 2018a) compared to rodents. Furthermore, a recent study demonstrated that in humans there is considerable overlap between the peptidergic and non-peptidergic markers CGRP and P2X3R respectively, markers which in rodents label distinct populations of DRG neurons (Shiers et al., 2020), thus suggesting that the molecular identities of sensory neurons might also be different in larger animals compared to rodents

Additionally, the longer life span of large animals enables longitudinal studying of both the early stages of arthritis, which is rather difficult in small animal models with a short initial phase and in humans where it goes unnoticed, as well as the long-term effects of interventional therapeutic use. Finally, it is possible to evaluate the safety and efficacy of new therapies in naturally occurring arthritis, usually found in large animals such as horse and dog, before advancing to human clinical trials (Koch and Betts, 2007). Although this discussion has focused on the possible benefits to human medicine of studying large animals, cases of naturally occurring arthritis in large animal species contribute a considerable veterinary burden (Anderson et al., 2018a) and thus more holistic study of arthritis in these animals themselves will likely provide beneficial clinical insight to veterinary practice, as well as the potential translational benefits to human pain therapeutics. However, several factors contribute to the current limited use of large animals in pre-clinical pain research as discussed below.

## 3 Limitations of large animal research

The major limitation most researchers face when considering the use of large animals in arthritic pain research, is the significantly higher cost associated with their housing and upkeep, both with regard to the facilities required for animal husbandry and lifespan. Secondly, there is the ethical question of using ‘higher’ species. For example, in the United Kingdom, use of animals in research is governed by the Animals (Scientific Procedures) Act 1986 Amendment Regulations 2012 and in applications to the Home Office to work with animals it is necessary to provide an explanation as to “why no other species is either suitable for the purpose or practically available” when considering the use of cats, dogs, primates and Equidae. Lastly, there is also the technical question surrounding the expertise required for *in vivo* study, as well as harvesting and culture of neurons/non-neuronal tissues required for *in vitro* analysis.

Compared to some large animal species, sheep and goats have a lower maintenance cost, are easily handled and are commonly used in arthritis research. However, since both sheep and goats are ruminants, comparing the pharmacokinetics and efficacy of experimental oral therapeutics to what might be observed in non-ruminant humans is particularly difficult. Although human joints are more similar to those of large animals than those of rodents, differences do still exist because, unlike humans, these animals are quadrupeds. For example, in dogs, total joint forces are split 60:40 between forelimbs and hindlimbs and thus the manifestation of hindlimb reduced load bearing in arthritis might be less pronounced in dogs (Meeson et al., 2019). Additionally, the trochlea of the distal femur is deeper in quadrupeds (Little et al., 2010). However, the major limitation to conducting studies in large animals is the lack of research tools. For example, it is difficult to obtain commercially available molecular biology reagents (e.g. validated polymerase chain reaction (PCR) primers, antibodies etc.) specific for large animal species to perform and analyze large scale genomic experiments in these species. Immunohistochemical analysis is further complicated by the fact that large animals, such as sheep and goats, are often used for the production of secondary antibodies, but such antibodies could not be easily employed for probing tissue in sheep and goat respectively.

The following sections will discuss the most commonly used large animals in arthritis research (See Table 1 for a summary of these models and key findings).

**Table 1:** Large animal models of arthritic pain.

Model	Large animals	Key features	Rodent equivalent? (Y/N)
Naturally occurring arthritis	Horse (Coppelman et al., 2019, Mariñas-Pardo et al., 2018; C. W. McIlwraith et al., 2012; Pujol et al., 2018)Dog (Alves et al., 2020, Carter et al., 1999, Malek et al., 2020, Moreau et al., 2014, Riley et al., 2016)Pig (Kreinest et al., 2016, Macfadyen et al., 2019)Monkey (Carlson et al., 1994, Rothschild et al., 1997)	Behavior: Clinical signs of lamenessAppearance: Inflamed (for inflammatory arthritis)Pathology: anterior cruciate ligament deficiency; cartilage erosion; synovium thickening and fibrosis; osteophytes formation; subchondral bone thickening and neovascularisationMolecular: Proteoglycans and type II collagen loss in cartilage	N, but occurs in transgenic animals (Christensen et al., 2016, Staines et al., 2017)
Degeneration-focused models of arthritis			
Monosodium Iodoacetate (MIA) induced arthritis	Pig (Uilenreef et al., 2019, Unger et al., 2018)Dog (Budsberg et al., 2019, Goranov, 2012, Pomonis et al., 2018)	Behavior: Lameness; increased asymmetric weight bearing;Pathology: cartilage necrosis and discoloration; synovial membrane thickening; subchondral bone necrosisMolecular: Increased pro-inflammatory cytokine expression profile in synovium	Y (Harvey and Dickenson, 2009, Udo et al., 2016)
﻿Osteochondral chip fragment model	Horse (Broeckx et al., 2019, Frisbie et al., 1997, Knych et al., 2017)	Behavior: LamenessPathology: Subintimal hyperplasia and fibrosisMolecular: Inflammatory genes expression change in synovial fluid; structural genes (collagen and aggrecan) expression change in cartilage	N
Osteochondral/Chondral defect induced arthritis	Horse (Niemelä et al., 2019, Salonius et al., 2019, Virén et al., 2012)Sheep (Crovace et al., 2019, Filardo et al., 2018, Newell et al., 2018, Olive et al., 2020, Pingsmann et al., 2005, Yucekul et al., 2017)Dog (Shortkroff et al., 1996, Zhang et al., 2018)Pig (Cunniffe et al., 2017, Pérez-Silos et al., 2019)	Behavior: Reduction in free movement as assessed by telemetryPathology: Fibrous and bone tissues at defect site; Subchondral bone pathologiesMolecular: Proteoglycan depletion in cartilage; increased expression of IL-6, IL-7, and TNF-α in synovium	Y (Matsuoka et al., 2015)
Meniscus injury induced arthritis	Sheep/Goat (Burger et al., 2007, Cake et al., 2013, Delling et al., 2015, Murphy et al., 2003, Song et al., 2014)Dog (Carlson et al., 2002)Pig (Otsuki et al., 2019)Monkey (Lutfi, 1975)	Behavior: Lameness; persistent gait abnormalityPathology: Cartilage erosion; Moderate osteophyteMolecular: Proteoglycan loss in cartilage; increased cytokine expression profile in synovium	Y (Glasson et al., 2007)
Anterior ligament transection induced (ACLT) arthritis	Sheep/Goat (Al Faqeh et al., 2012, Atarod et al., 2014, Barton et al., 2019, Delling et al., 2015, Murphy et al., 2003, Song et al., 2014)Dog (Smith et al., 2002, Widmer et al., 1994)	Behavior: Kinematic changes in gaitPathology: Significant gross joint damage; Meniscal damage; Osteophyte formationMolecular: Increased expression of type II collagen in cartilage; decreased MMP-3 expression in synovium	Y (Xie et al., 2018)
Trans-articular load model (non-invasive)	Dogs (Lahm et al., 2005, Thompson et al., 1991)	Pathology: Subchondral fractures and microfractures, but intact ligaments and menisci	Y (Poulet et al., 2011)
Inflammation-focused models of arthritis			
Complete Freund’s adjuvant (CFA) induced arthritis	Horse (White et al., 1994)Sheep/goat (Deng et al., 2018)Dog (Haak et al., 1996)	Behavior: Severe lamenessPathology: inflammatory synovitis, pannus formationMolecular: notable infiltration of mononuclear cells in joint	Y (Chillingworth and Donaldson, 2003)
Collagen induced arthritis	Sheep (Abdalmula et al., 2014)Monkey (Korver et al., 2019)Pig (Lee et al., 2016)	Behavior: Clinical signs of lamenessAppearance: Joint swellingPathology: Synovium thickening; cartilage erosionMolecular: increased monocytes and lymphocytes count in synovial fluid; increased expression of TNF-α, IL-1β and VCAM-1 in synovium	Y (Brand et al., 2007, Pietrosimone et al., 2015)
Antigen induced arthritis	Pig (Naujokat et al., 2019, Vela et al., 2017)Sheep (Highton et al., 1997)	Pathology: synovial inflammation; cartilage surface alteration; chondrocyte clusters formationMolecular: increased expression of IL-1β, IL-6, TNFα and VEGF in synovium	Y (Brackertz et al., 1977)
Amphotericin induced synovitis-arthritis	Horse (Barrachina et al., 2016, Suominen et al., 1999)Pig (Whalin et al., 2016)	Behavior: Increased lamenessAppearance: Joint effusion and local joint heatPathology: Cartilage discoloration, fibrillation and erosions; synovium subintimal changesMolecular: increased white blood cell count and haptoglobin expression in synovial fluid	Y (Lee et al., 2008)
Carrageenan induced arthritis	Horse (Owens et al., 1996)Dog (Hansen et al., 1990, Søballe et al., 1991)Pig (Uruchurtu Marroquin and Ajmal, 1970)	Behavior: Increased lamenessAppearance: Local joint heatPathology: Increased synovium volumeMolecular: increased PGE_2_ expression in serum	Y (Hansra et al., 2000, Ikeuchi et al., 2009)
Lipopolysaccharide (LPS) induced arthritis	Horse (Banse and Cribb, 2017, Cokelaere et al., 2018, Neuenschwander et al., 2019, Ross et al., 2012)	Behavior: Severe lamenessAppearance: Joint swellingPathology: SynovitisMolecular: Appearance of Serum amyloid A in blood and synovial fluid; increased white blood cell count and total protein in synovial fluid; increased PGE_2_ expression in serum	Y (Tanaka et al., 2006)

## 4 Naturally occurring and models of arthritis in large animals

### 4.1 Naturally occurring

As mentioned above, large animals (e.g. dogs, horses, pigs and rhesus monkeys) are, similar to humans, prone to naturally occurring arthritis. Pain caused by arthritic conditions is a major veterinary burden with significant cost to the global economy, the equine industry being the flagship example. Lameness occurs in ~ 60% of horses, most cases of which are attributed to naturally-occurring OA and cost millions of US dollars to the global economy because the equine business is a multibillion dollar industry (Conners and Feldman, 2009, McIlwraith et al., 2012). Naturally occurring arthritis is useful for identifying mechanisms associated with various stages of arthritis and for investigating the disease in a similar environment to which humans are exposed to. For example, dogs are human companion animals and, as in humans, show an increased risk of OA with age and obesity with an annual prevalence rate of 2.5% in UK veterinary primary care practices (Meeson et al., 2019). Recently, 75% of > 80 week old commercial pigs (female, Large white × Landrace × Duroc) were also observed to develop arthritis naturally with associated pain behavior (Macfadyen et al., 2019), thus opening doors for more in-depth research in this species, alongside dogs and horses. Although naturally occurring arthritis is ideal for studying clinical disease progression, the major disadvantage is cost because the animals have to be monitored for a prolonged period of time. In addition, there are significant individual variations in arthritis presentation, as well as the requirement of a large number of animals to achieve sufficient statistical power.

### 4.2 Degeneration-focused models of arthritis

Given the propensity of naturally occurring arthritis in both humans and non-humans to be OA, a number of degeneration-focused (i.e. OA-like) models have been developed in large animals. Experimentally, OA can be induced by numerous methods, including: injection of chemical substances like monosodium iodoacetate (MIA), surgical damage to the joint, joint destabilization, and by impact trauma on the joint surface. Among chemically induced OA models, MIA injection into the joint is most commonly used and acts by inhibiting glyceraldehyde-3-phosphate dehydrogenase (GAPDH, an enzyme involved in glycolysis), which leads to the death of chondrocytes and has proven useful for understanding OA pain mechanisms (Combe et al., 2004, Samvelyan et al., 2020). The MIA model of OA has been successfully induced in pigs and dogs, as evidenced by both lameness and structural changes in the joints being observed in these animals following MIA administration (Budsberg et al., 2019, Uilenreef et al., 2019). Joint damage models, for example osteochondral fragment models, are well described in the horse, whereas destabilization of the joint is more commonly described in ruminant and dog models. Joint destabilization can be achieved surgically in a reproducible manner, making such procedures the models of choice for understanding the immediate response to altered joint biomechanics and the subsequent chronic stages of arthritis. Of the models that have been developed, anterior cruciate ligament transection (ACLT), meniscectomy and medial meniscal transection are the most commonly used surgical approaches that have been shown to induce arthritis (Table 1). However, given the invasive nature of inducing joint destabilization, such models may not be particularly useful to study the early stages of OA development that is not associated with traumatic injury (Malfait et al., 2013). Correspondingly, non-invasive models have been developed, in dogs, where OA is produced by transarticular impact without the requirement of invasive surgical intervention (Lahm et al., 2005).

### 4.3 Inflammation-focused

Inflammation is a common clinical symptom for both OA and RA, and is often accompanied by pain, consequently, several inflammation-based models of arthritis have also been developed. Such models can also provide important insights into naturally occurring RA that has been observed in dogs (Carter et al., 1999) and monkeys (Rothschild et al., 1997), similar to naturally occurring RA in humans, i.e. IgM rheumatoid factors are upregulated in sera and synovial fluid. Among the induced animal models of arthritis, perhaps the most commonly used is injection of complete Freund’s adjuvant (CFA, a paraffin oil emulsion of heat killed mycobacteria, usually *Mycobacterium tuberculosis*) that causes both acute and chronic inflammation, characterized by leukocyte infiltration, synoviocyte hyperplasia, pannus formation and pain. Intra-articular CFA injection has been successfully used to induce arthritis in horses, dogs and sheep as evidenced by persistent lameness for ~ 2 weeks (Deng et al., 2018, Haak et al., 1996, White et al., 1994). The major criticisms of this model are firstly, that it bypasses the autoimmune component of RA and secondly, that it causes milder cartilage damage compared to human RA, and therefore the collagen-induced arthritis (CIA) model was developed in which type II collagen is administered in combination with CFA. To generate CIA, large animals are first sensitized with collagen type II emulsified in CFA by sub-cutaneous injection, following which arthritis is induced by subsequent injection of collagen type II (Abdalmula et al., 2014); however, collagen based models engage only a subset of T helper (Th) cells that are involved in human RA (Stoop et al., 2013). In addition to collagen, other antigens such as bovine serum albumin and ovalbumin have also been utilized to induce arthritis in large animals and are classified as antigen-induced arthritis (AIA) (Highton et al., 1997, Naujokat et al., 2019). Alongside the above mentioned chronic models of arthritic pain, various acute models exist, whereby joint inflammation and pain are induced by intra-articular injection of an inflammatory substance (e.g. amphotericin, carrageenan or lipopolysaccharide) that causes similar behavioral changes to those observed in chronic models, albeit for a more limited time frame (~48 h), a significant benefit being reduced time and cost to the investigator (Neuenschwander et al., 2019, Owens et al., 1996).

Arthritic pain in the above-mentioned models can be studied behaviorally by measuring several outcomes *in vivo* or mechanistically at a cellular level *in vitro*. The two main categories of behavioral pain measures are evoked and spontaneous pain measures. Evoked pain behaviors measure the reaction of an animal to exogenous stimuli, e.g. withdrawal threshold to mechanical stimulation of the hind paw; however, it is controversial whether these reflexive behaviors reflect true “pain” (Deuis et al., 2017). In contrast, non-reflexive, spontaneous pain behaviors might better recapitulate the human experience of persistent, ongoing pain that decreases quality of life. However, one important factor to note is that by definition pain has a sensory and emotional component, and hence use of the term here is anthropomorphic owing to our inability to know the true emotional state of any non-human animal, and hence we can only comment about “pain-like” states in animal models.

The most commonly assessed non-reflexive behavioral outcome in large animal models of arthritis is lameness. Lameness is historically scored visually, based upon previously established criteria and by the stride length of an animal when walking on sand (Thomsen et al., 2008, White et al., 1994). More recently, however, technologically advanced systems have been developed where in-depth quantitative kinematic gait analysis can be conducted by implanting an instrumented spatial linkage device on bones (Barton et al., 2019) or by analysis using a motion capture camera while an animal walks on a treadmill (Bockstahler et al., 2009, Sanchez-Bustinduy et al., 2010). Simpler methods have also been developed to quantify force applied by each limb using pressure mat systems (Uilenreef et al., 2019) or force plates on treadmills (Belshaw et al., 2016). Additionally, telemetry based analysis of distance travelled in freely moving animals has also shown promise for evaluating pain behavior in sheep (Newell et al., 2018). Besides lameness, inflammation is another widely assessed *in vivo* outcome in arthritis models, although it should be noted that although inflammation and pain often occur concomitantly, inflammation can occur in the absence of pain and vice versa (Bedson and Croft, 2008, Salaffi et al., 2018). Similar to lameness, inflammation is primarily assessed by visual scoring according to previously standardized guidelines and/or by using Vernier’s calipers (Abdalmula et al., 2014, Lee et al., 2016). Joint heat is another measure of inflammation due to the fact that increased temperature often accompanies joint swelling and this can be recorded using an infra-red laser thermometer (Barrachina et al., 2016). In large animals, inflammation can also be assessed by imaging technologies such as X-ray radiography, computer tomography and magnetic resonance imaging (Crovace et al., 2019, Lee et al., 2016, Salonius et al., 2019). For specifically measuring pain in a non-reflexive manner, grimace scales have been developed for horses (Dalla Costa et al., 2014), sheep (Häger et al., 2017) and pig (Viscardi et al., 2017), although these have not yet been widely utilized in arthritis research. In addition to the above described non-reflexive outcome measures, a limited set of reflexive pain behavior can also be measured by manually flexing/palpating the joint until the animal shows sign of discomfort (Lee et al., 2016, White et al., 1994).

## 5 *In vitro* models to study peripheral mechanisms of arthritic pain

**Fig. 2:**
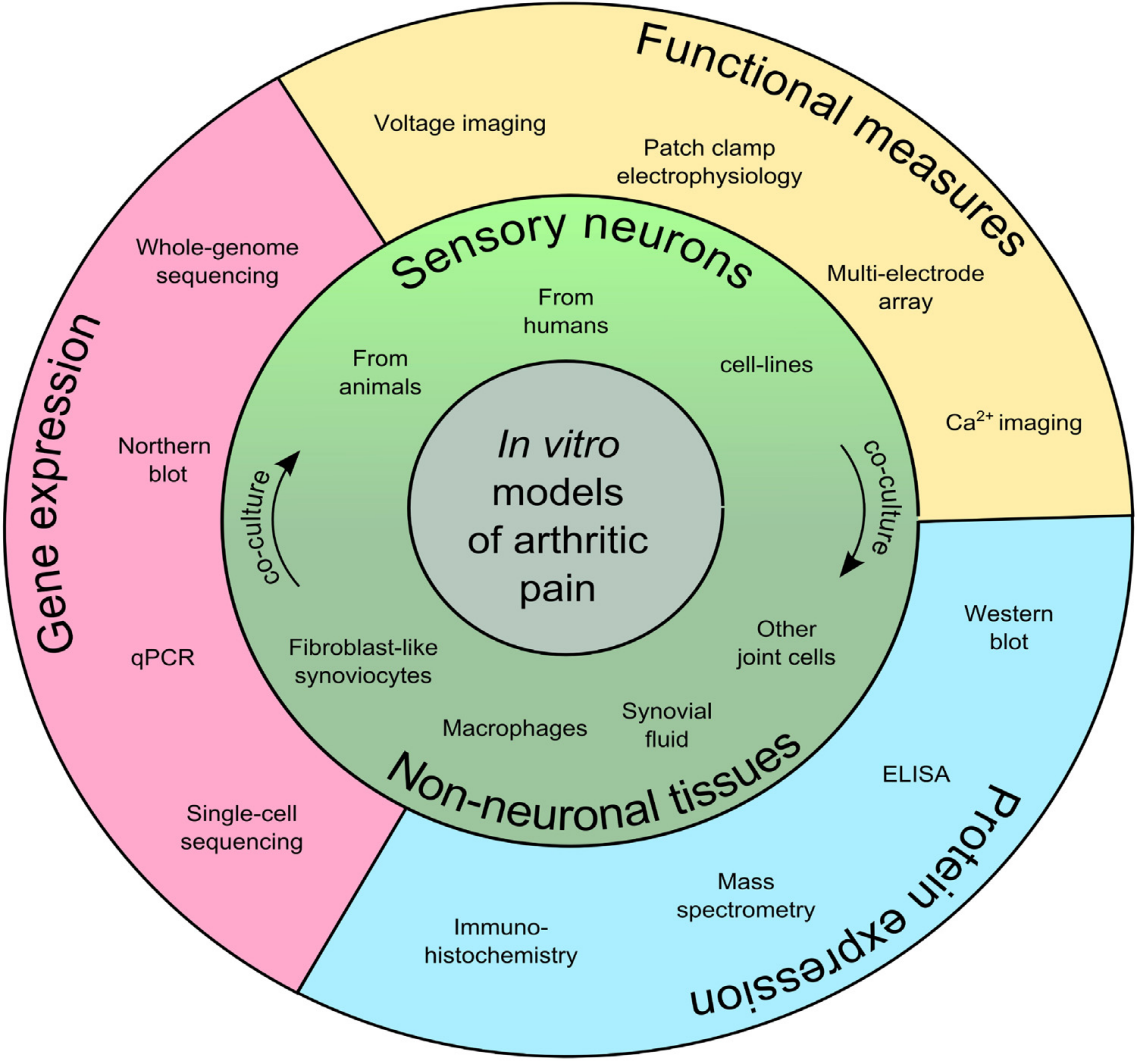
Pictorial representation of existing *in vitro* models to study and assess mechanisms of arthritic pain.

Although considerable progress has been made in the field to develop large animal models of arthritis and assessment of behavioral and structural outcomes, the understanding of cellular mechanisms of arthritic pain from *in vitro* analysis in these animals is surprisingly limited. The rationale for developing *in vitro* models of arthritic pain is based on the philosophy of reductionism (Kaiser, 2011), such that a complex disease like arthritis can be studied at the cellular and molecular level, away from confounding systemic effects. Even so, an *in vitro* model must still show some manifestation of the *in vivo* phenotype of interest to facilitate understanding of disease mechanisms and discovery of drug targets. Consequently, multiple *in vitro* models of pain and assays to test these models have been developed. The major strategy utilized in these models is to harvest tissues from animals undergoing a model of arthritis (primarily from rodents) or from human biopsy, surgery or biobank samples. The technological toolbox and validated techniques available to pain researchers working with rodents is currently much more diverse and efficient, than what is available and validated for researching arthritis pain mechanisms in large animals. The following paragraphs review the *in vitro* models and assays commonly used to study arthritic pain in rodents, with a focus on those which we believe can be adapted in large animal research (Summarized in Fig. 2).

### 5.1 Drg neurons

Each DRG contains cell bodies of primary sensory neurons that innervate the periphery, apart from the head and neck that are innervated by sensory neurons arising from the trigeminal ganglia. Somatosensory information from the periphery is first processed by the primary sensory neuron, which relays the information to the CNS, and hence DRG neurons act as the gatekeeper between the PNS and CNS (St. John Smith, 2018). DRG neurons are pseudo-unipolar, one branch extending to the peripheral organ and the other branch synapsing with neurons in the dorsal horn of the spinal cord. In addition to DRG neurons being equipped with the receptors and ion channels required for detecting noxious stimuli and thus being critical in the pain pathway, they are relatively easy to dissect and culture, which makes DRG neurons an important *in vitro* model for studying mechanisms of pain. Experimentally, DRG neurons have been studied *in vivo*, *ex vivo* and *in vitro*, with acutely dissociated neuronal cultures from control and diseased rodents *in vitro* being the most commonly used setup in recent years (Melli and Höke, 2009); mouse DRG neuron cell lines are also available, but these are typically less physiologically relevant (Doran et al., 2015). The first AP recordings from rodent DRG neurons were conducted electrophysiologically *in vivo* in terminally anesthetized rats using sharp electrodes (Harper and Lawson, 1985, Ritter and Mendell, 1992). This technique enabled both morphological and functional characterization of mechanoreceptors based on their conduction velocity and site of innervation, as well as to record changes in these sensory neurons when an inflammatory agent was injected at the distal site. However, this system is technically challenging since a laminectomy has to be performed on an anaesthetized, live animal before recordings can be conducted; additionally, not all DRG neurons can be accessed using this technique. By contrast, DRG can be seeded as explants *in vitro* to perform experiments in a more controlled manner than *in vivo* (Gong et al., 2016). In explant cultures, the *in vivo* morphology of DRG and associated non-neuronal Schwann cells and macrophages is retained, features that are lost when using dissociated cultures (Melli and Höke, 2009). Since DRG explants grow nerve processes, the interaction between DRG axons and other cells/inflammatory mediators can be studied using Campenot chambers (Campenot, 1977). By contrast, although acutely dissociated DRG neuron cultures *in vitro* do not allow for the study of axons, they do offer the experimenter an unparalleled opportunity to characterize individual neuronal cell bodies, which have been shown to have largely similar properties to their terminals (Harper, 1991, Wangzhou et al., 2020a). Furthermore, acute DRG neuron cultures have emerged as robust *in vitro* models of pain since they reflect the hypothesized neuronal basis of pain in experimental animal models, such as changes in nociceptive gene expression and excitability. For example, in a rat AIA-induced ankle inflammation model, whole-cell patch clamp recordings from *in vitro* acutely cultured DRG neurons revealed increased excitability of joint neurons, which was consistent with the joint inflammation and mechanical hyperalgesia observed behaviorally in the affected limb (Qu and Caterina, 2016). Precise mechanisms of an inflammatory mediator’s effect on sensory neurons can also be elucidated in these cultures (von Banchet et al., 2005). In addition to the reasons described above, acutely cultured DRG neurons enable whole-cell patch clamp recording of individual retrograde-labelled neurons from a peripheral organ, which is not possible in a more intact preparation.

Although there is a substantial body of literature on the expression profile of nociceptive genes and neuronal excitability of DRG neurons in arthritic pain, limited information is available on how arthritis specifically modulates joint-innervating DRG neuron gene expression and excitability. The importance of studying joint-specific disease mechanisms is highlighted by the high level of heterogeneity of DRG neurons (Zeisel et al., 2018) and the demonstration of specific subpopulations innervating the colon (Hockley et al., 2019), i.e. site of innervation is important. Data from our lab have demonstrated that the AP threshold of retrograde-labelled knee-innervating DRG neurons is lower in ipsilateral neurons than in contralateral neurons in a mouse model of inflammatory arthritis (Chakrabarti et al., 2018) and, furthermore, specifically tuning down the excitability of joint-innervating neurons using adeno-associated virus chemogenetic tools can provide pain relief (Chakrabarti et al., 2020c). Given the utility of DRG neurons in studying pain, efforts have been made to characterize human DRG neurons derived from pain pathologies, although not yet in the field of arthritis (Haberberger et al., 2019). This is perhaps because arthritis has a high incidence rate in the population and hence the likelihood of obtaining “control” human DRG (i.e., with no known joint disease) is low. Therefore, identifying a large animal model that reproducibly simulates human arthritis pain features, and thus likely the underpinning pain mechanisms, would be a very useful and relevant research tool.

Comparative analysis of human and rodent DRG neurons has highlighted important differences. Firstly, human DRG neurons are larger than those of rodents (range of soma size: 12–40 µm in mouse vs. 20–100 µm in humans) (Davidson et al., 2014, Rostock et al., 2018b, Silos-Santiago et al., 1995), but are similar to DRG neurons of large animals like those of sheep ((Domenico Russo et al., 2010b), range of soma size: 20 – 70 µm unpublished observation by the authors – see representative image in Fig. 1). Secondly, expression and function of some receptors important in pain pathologies are differentially regulated in humans compared to mice (Ray et al., 2018, Shiers et al., 2020, Wangzhou et al., 2020a). For example, in human DRG neurons, the voltage-gated sodium channel (Na_V_) 1.8 blocker A-803467 is much less effective at blocking Na_V_-mediated currents in human DRG neurons than in rat DRG neurons, suggesting that Na_V_ blockers with efficacy in rodents might not translate to clinical pain relief in human diseases due to different expression levels (Zhang, et al., 2017). The feasibility of obtaining DRG from large animals has been demonstrated in many species including horses (Russo et al., 2010a), sheep (Deng et al., 2018, Dudek et al., 2017, Domenico Russo et al., 2010b), pigs (Jonas et al., 2015, Klusch et al., 2018, Kozłowska et al., 2017, Obreja et al., 2008, Sandercock et al., 2019) and dogs (Ganchingco et al., 2019, Schwarz et al., 2019), providing proof-of-concept that DRG neurons from large animals can be utilized as *in vitro* models for arthritis pain.

### 5.2 Non-neuronal tissues

The previous section emphasized the importance of DRG neuron hyperexcitability in chronic pain conditions like arthritis. However, hyperexcitability is often mediated by neuronal exposure to an inflammatory environment produced by non-neuronal cells and thus investigating these non-neuronal cells is also important for understanding arthritis pain mechanisms and identifying new therapeutic targets. Indeed, with regard to the inflammatory environment of arthritis, it should be noted that exposure of knee-innervating neurons to synovial fluid from OA patients in pain causes neuronal sensitization (Chakrabarti et al., 2020b). The on-going pathology of both RA and OA register as tissue damage in the body, which leads to triggering of innate immune responses and recruitment of a variety of cells through damage associated molecular patterns (Miller et al., 2019, Sokolove and Lepus, 2013). A non-neuronal cell of significant interest in arthritis is the fibroblast-like synoviocytes (FLS), a cell type thought to be one of the key effectors of arthritis and can be maintained in culture for prolonged period of time (Bartok and Firestein, 2010). Indeed one of the mechanisms of action of the disease-modifying anti-rheumatic drug methotrexate is reduction in FLS proliferation (Lories et al., 2003) and a reduction in FLS number leads to a reduction in the levels of inflammatory mediators that they secrete and which drive arthritic pain (Sokolove and Lepus, 2013). *In vitro* analysis of FLS has mostly focused on gene expression and protein assessment of factors released into the culture medium to show that cytokine stimulated rodent FLS or human arthritic joint-derived FLS show upregulated pro-inflammatory gene expression and cytokine release (Hong et al., 2018, Jones et al., 2016, Kawashima et al., 2013); similar results have been obtained in some rodent models, such as K/BxN (Hardy et al., 2013) and AIA (von Banchet et al., 2007). In addition, whole-cell patch clamp performed on rodent FLS has identified the presence of various voltage-gated K^+^ and Ca^2+^ channels (Clark et al., 2017, Haidar et al., 2020). However, these results need to be verified in human-derived FLS and the effect of inflammatory mediators on these channels investigated.

In addition to FLS, T cells, B cells and macrophages have also been studied to understand their role in arthritic pain. In brief, investigation of T cells has identified a range of distinct subtypes based upon their cytokine secretion profile (Raphael et al., 2015), which play distinct roles in arthritis by sensitizing joint nociceptors. Additionally, in a co-culture study it was found that IL-21 producing T cell mediated joint destruction occurs because these cells stimulate FLS to secrete matrix metalloproteases, which in turn contribute to joint destruction (Lebre et al., 2017), thus underlining the importance of cross-talk between non-neuronal cell types. B cells on the other hand have been shown to inhibit osteoblast formation in RA through activity of the cytokines CCL3 and β), tumor necrosis factor α (TNF- α) (Sun et al., 2018). Macrophages are another heterogeneous cell class that plays prominent inflammatory roles in both RA and OA (reviewed in (Udalova et al., 2016, Wu et al., 2020)), although their lineage characteristics might be lost in culture, thus limiting extensive studies *in vitro* (Chamberlain et al., 2015).

A handful of studies have also attempted to study non-neuronal cells in combination with DRG neurons to understand the inflammation-pain axis (Massier et al., 2015, von Banchet et al., 2007). For example, in neuron-macrophage co-cultures, lipopolysaccharide (LPS)/IFN- γ stimulated macrophages were observed to increase CGRP release from DRG neurons in both direct (cells cultured together) and indirect (neurons only come into contact with macrophage-derived soluble mediators) co-cultures, thus demonstrating the importance of inflammatory mediators in neuronal activation. FLS co-cultured with DRG neurons have also been shown to increase excitability and modulate the mechanosensory micro-environment of neurons (Chakrabarti et al., 2020a, Ita and Winkelstein, 2019). Looking to the future, the field of co-culture study has recently received a boost with the development of microfluidics techniques (Vysokov et al., 2019), which can also be useful to study arthritic pain *in vitro*.

With regard to examining the roles of non-neuronal cells in large animal models of arthritis, successful culture of FLS from synovium punch or synovial fluid has been conducted in horses (Ghasemi et al., 2017, Warnock et al., 2014), sheep (Smith et al., 2008) and dogs (Pelletier et al., 1997). Macrophages and lymphocytes have also been cultured from large animals such as, sheep, pigs and dogs (Beya et al., 1986, Herrmann et al., 2018, Jungi et al., 1992, Saalmüller et al., 1994). These results thus demonstrate that non-neuronal/neuronal co-culture studies can also be set up with cells derived from large animals. Consequently, such techniques could be employed more widely in the pain research field to better understand inflammatory pain based upon the points made earlier regarding the benefits of large animal use in general, as well as specific differences in immune system between humans and mice. For example, laboratory mice show clear dichotomy in polarization of Th1/Th2 cells when stimulated with specific cytokines (such as, IL-4 stimulates Th1 and IFN- γ stimulates Th2), while cattle and humans appear not to strictly adhere to this paradigm (Estes and Brown, 2002, Guzman and Montoya, 2018, Mestas and Hughes, 2004). Since the underlying motivation of *in vitro* analysis is to better understand the cellular and molecular pathways generating pain in arthritis, multiple assays have been developed to enable interrogation of cellular function as described in section 5.3 below.

### 5.3 *In vitro* assays to understand arthritic pain

The *in vitro* assays for investigating cellular basis of arthritic pain can be largely divided into three categories that seeks to assess: 1) gene expression changes, 2) protein expression changes, and 3) functional changes. It is important to separate these categories to understand disease mechanisms because their interactions are not always predictable.

#### 5.3.1 Gene expression

Gene expression studies enable assessment of how different genes might contribute to a particular pathology and are typically conducted by comparing differential expression patterns in healthy vs. diseased tissues. One of the first modern gene expression assays to be developed that is still widely used today, was the quantitative PCR (qPCR). In this technique, primers are used to amplify a specific region of DNA. One method for quantifying the amount of starting material is to measure the fluorescence emitted by a fluorophore that is initially attached to the primers and kept non-fluorescent by the presence of a quencher which is cleaved off as the primer becomes incorporated into the DNA product freeing the fluorophore as it becomes separated from the quencher leading to an increase in fluorescence (San Segundo-Val and Sanz-Lozano, 2016). qPCR has helped identify genes that are upregulated in the synovium in the MIA model of joint pain in mice, hence providing useful insights into disease mechanisms in OA (Dawes et al., 2013), although follow up work is always required to determine the impact of changes in gene transcription with regard to disease pathology and pain sensation. Although PCR based techniques are easy and fast to conduct, their primary drawback is that they are of low-throughput and do not allow for unbiased probing of differential gene expression. By contrast, microarray-based transcriptomics enable low cost, high throughput studies for a limited set of genes using the principle of hybridization of cDNA with oligonucleotides (Starobova et al., 2018). Application of microarray analysis to mRNA extracted from joints of naturally-occurring RA mouse models has identified pathogenic gene clusters, such as chemokine genes and histocompatibility genes (Fujikado et al., 2006). This result was further validated using Northern blot, a technique where denatured RNA is loaded in an agarose gel and separated by electrophoresis to assess gene expression.

The field of gene expression studies has been revolutionized in recent years with the advent of RNA-sequencing, whereby whole transcriptome analysis, either from tissues or single cells, enables unbiased analysis of differential gene expression. The focus of transcriptomics in pain research has largely been on DRG neurons and large databases have been generated to compare between different species and between healthy and painful conditions (Megat et al., 2019, North et al., 2019, Ray et al., 2018). With the recent advances in bioinformatic tools it was also possible to combine these datasets to construct interactomes of neuronal and non-neuronal communications (Wangzhou et al., 2020b). Although most of these studies were conducted with rodent and human samples, recently a whole DRG RNA-sequencing study in sheep and goat models of inflammatory pain (CFA in the foot) and a microarray analysis of tail amputated pigs have identified clusters of genes associated with inflammatory and neuropathic pain (Deng et al., 2018, Sandercock et al., 2019). RNA-sequencing data from canine DRG neurons have also been obtained in a cross-species (rat, dog and human) study demonstrating the efficacy of ablating TRPV1 nerves in providing pain relief (Sapio et al., 2018).

Additionally, single cell transcriptomics has been instrumental in arthritis and pain research by identifying clusters of sensory neurons (Hockley et al., 2019, Hu et al., 2016, Usoskin et al., 2015, Zeisel et al., 2018), synovial fibroblasts (Croft et al., 2019, Zhang et al., 2019) and chondrocytes (Ji et al., 2019), but at the time of writing there has not been a single-cell RNA-sequencing analysis that specifically examines how joint-innervating neuron gene expression changes in arthritis in any species, but such a study would clearly provide important insight into pain mechanisms and potential drug targets in arthritis.

#### 5.3.2 Protein expression

Although gene expression analysis provides insights into disease mechanisms, gene expression does not always translate to protein expression. Therefore, several assays that measure protein expression have been developed. A widely used antibody based, semi-quantitative technique for measuring protein expression is immunohistochemistry which is regularly used in the pain field and enables the investigator to observe protein expression on a cell-by-cell basis (Cregger et al., 2006). Two dimensional electrophoresis is another semi-quantitative method that involves electrophoresis, staining, fixing and densitometry, but it does not provide the cellular level of detail that immunohistochemistry can provide (Greenbaum et al., 2003). More quantitative methods have also been developed, the simplest of which is the enzyme linked immunosorbent assay (ELISA) where antibody-conjugated enzyme activity is monitored to measure protein expression, usually of a mediator released into the extracellular environment, e.g. a cytokine or neuropeptide (Engvall, 1980). Mass spectrometry (MS) is a more sophisticated way of quantifying proteins and has become popular in pain research in recent years (reviewed in (Wood et al., 2018)). In this technique protein extracts from tissues are cleaved into short peptides and separated by chromatography before being analyzed in a mass spectrometer. Using MS on DRG protein extracts from pre-clinical murine models has provided useful insights in chronic pain (Rouwette et al., 2016); and proteomic analysis of synovial fluid taken from arthritis patients has verified known proteins (e.g. matrix metalloproteases) as well identified as novel proteins (e.g. thymidine phosphorylase, reticulon 4 receptor-like 2) involved in the disease mechanism (Balakrishnan et al., 2014). Additionally, quantitative methods of identifying components of ion channel protein complexes, such as Na_v_s, have also been developed in recent years (Kanellopoulos et al., 2018, Rees et al., 2017).

The field of large animal research has used, and continues to rely mostly on, histological analysis of joints using a modified Mankin or O’Driscoll scoring system (Abdalmula et al., 2014, Haak et al., 1996, Naujokat et al., 2019, Newell et al., 2018), often accompanied by protein level immunoprecipitation of inflammatory mediators such as prostaglandins E2 (PGE2), IL-6 and IL-1 β, TNF- α in the serum, synovial fluid and/or synovium tissue (Barrachina et al., 2016, Neuenschwander et al., 2019, Owens et al., 1996). A handful of studies have also revealed expression of pain-related proteins (e.g. CGRP and substance P) in the DRG neurons of sheep, pigs, horses and dogs (Hoover et al., 2008, Obreja et al., 2008, Domenico Russo et al., 2010b, Russo et al., 2010a, Tamura et al., 1996). However, how protein expression changes in the context of pain and specifically arthritic pain, remains to be elucidated. The promise of this strategy has been demonstrated in a study where immunohistochemical analysis of healthy and laminitic horses showed increased expression of neuronal injury marker, ATF3, and neuropeptide Y in DRG neurons indicating a likely neuropathic contribution to pain in laminitis (Jones et al., 2007).

Results from these studies suggest that generating omics datasets from large animals and integrating them with the high-resolution and varied datasets already available from mouse and humans could boost the field of pain research. However, the current data rich era of cross-species proteomics and transcriptomics highlights the need for bioinformatics in pain research, as well as development of online platforms for sharing data collected by different labs to enable researchers to compare datasets (e.g., http://rna-seq-browser.herokuapp.com/, https://bbs.utdallas.edu/painneurosciencelab/sensoryomics/, accessed on 10/4/2020) and identify key pain mechanisms (Jamieson et al., 2014, Platzer et al., 2019).

#### 5.3.3 Functional assays: Electrophysiology and voltage imaging

Although transcriptomics and proteomics can help identify promising targets for pain research, functional tests are essential for assessing their actual contribution of a target to the disease. This is largely because, in addition to changes in gene, and thus potentially also protein, expression levels, post-translational modification of numerous ion channels occurs, including many involved in nociceptor function, such as TRPV1 and Na_V_s, which can also have a significant impact on nociceptor excitability, but would not be picked up by simple expression analysis (Hall et al., 2018, Laedermann et al., 2015). Additionally, functional assays can form an efficient bridge for understanding peripheral pain mechanisms between *in vitro* and *in vivo* technologies, because of the development of *ex vivo* and semi-intact setups. For example, electrophysiological recordings from *ex vivo* skin-innervating nerve endings (Walcher et al., 2018) can help reconcile findings from *in vivo* behavioral assays (such as von Frey) with detailed *in vitro* cellular insights from DRG neurons. This desire to probe the nociceptive circuitry from the peripheral nerve endings to the spinal cord has also led to the development of a semi-intact preparation in which the skin through DRG to spinal cord is intact and recordings can be performed at multiple sites throughout this circuit (Hachisuka et al., 2016).

The two most commonly used cellular functional assays in pain research are electrophysiology to measure changes in current or voltage across the cell membrane in response to different stimuli, or, alternatively, fluorescent dyes that enable measurement of the intracellular [Ca^2+^] as a readout of cellular excitation can be used.

Measurement of voltage changes across nerve fibers began with the seminal work of Hodgkin and Huxley where they recorded intracellular APs in squid giant axons using electrodes (Hodgkin and Huxley, 1939). Their work also led the way for the groundbreaking development of whole-cell patch clamp techniques by Neher and Sakmann, where a cell could be held at any command voltage, to record current and voltage either across a whole cell or single ion channels. Multiple conformations of the patch clamp technique enable recording the activity of ion channels when stimuli are applied to the outside (whole cell recording and outside out patch) or inside (inside out patch) of the cell membrane (Sakmann and Neher, 1984), achieved by appropriate maneuvering of the electrode. Electrophysiological techniques have provided many fundamental insights about inflammatory pain, such that the excitability of DRG neurons is observed to increase when comparing neurons isolated from healthy animals to those isolated following an inflammatory insult in cats (Xu et al., 2000), rats (von Banchet et al., 2000), guinea pig (Djouhri and Lawson, 1999) and mice (Belkouch et al., 2014). Correspondingly, *in vivo* recordings from rat joint afferents have shown increased neuronal excitability after PGE_2_-induced inflammation (Grubb et al., 1991). Furthermore, single channel recordings have demonstrated the sensitization of mechanosensitive ion channels in DRG neurons isolated from mice with OA (He et al., 2017).

Although patch clamp is a very precise way of understanding ion channel function, it is relatively low throughput, labor intensive and requires substantial expertise of the experimenter. To increase the throughput of this assay multi-electrode arrays have been used that can simultaneously record from multiple neurons (Mis et al., 2019). In order to bypass the manual expertise, automated micropipette based platforms have been developed that capture and seal cells in suspension and can produce results at a higher throughput (reviewed in (Annecchino and Schultz, 2018)), but such devices are not generally suited to measuring the function of ion channels in DRG neurons that grow neurites in culture and whose function is modulated by the surface they are grown on. Additionally, there are currently no automated patch clamp platforms for assessing mechanical stimuli on DRG neurons. However, several ion channels important in pain pathologies have been studied in cell lines using this technique including Na_V_s, hyperpolarization activated cyclic nucleotide gated (HCN) and voltage-gated Ca^2+^ channels (Ca_V_s) (Payne et al., 2015, Swensen et al., 2012, Vasilyev et al., 2009). Overall, the relatively high throughput of these platforms makes them very useful for compound screening, but further development and cost optimization is necessary before automated patch clamp platforms replace the manual patch clamper in the lab.

The advantage of the patch clamp technique is that it provides direct access to neurons, however, it is also a disadvantage because direct contact with the neuron, even in perforated patch clamp technique where the aim is to minimize disruption of neuronal function, can change membrane properties and disrupt cytoplasmic content. Therefore, an ideal experiment would be to image changes in neuronal voltage in a high throughput manner (reviewed in (Bando et al., 2019a)). This can be achieved by loading voltage sensitive dyes into neurons and measuring the membrane potential especially in large neurons *in vitro*. *In vivo*, single cell resolution is difficult to achieve with voltage sensitive dyes and hence genetically encoded voltage indicators (GEVIs) have been developed. Technically this can be achieved by three different ways: coupling the voltage sensor to a fluorescent protein (e.g., ArcLight (Bando et al., 2019b)), using rhodopsin to act as both a voltage sensor and reporter (e.g., VARNAM (Kannan et al., 2018)) and lastly by using chemicals that activate GEVIs (e.g., HAPI-Nile (Sundukova et al., 2019)). However, imaging voltage in neurons is not without challenges, the most important ones being thinness of the membrane which demands high sensitivity chromophores, difficulty in specifically targeting the plasma membrane and photo-damage of the plasma membrane (Bando et al., 2019a).

Utilization of patch clamp electrophysiology in large animal research in the field of pain is largely unchartered territory. A PubMed search (conducted on 19/5/2020) with the terms “patch clamp mouse neuron pain” yielded 316 results, however, when the term mouse was replaced by sheep, dog or horse no results were found and only one article was found for pig (Note: a “NOT guinea” clause was added for pig and one result obtained for dog actually conducted the patch clamp experiments on rat DRG neurons). The study on porcine DRG neurons demonstrated the presence of a subclass of DRG neurons that are capsaicin responsive, but lacks HCN mediated currents, therefore suggesting analgesics targeting HCN might have restricted success in pigs (Obreja et al., 2008). Another study aiming to understand functional responses of porcine DRG neurons to the inflammatory agent NGF found release of CGRP from the neurons as well as neurite sprouting (Klusch et al., 2018). Therefore, although there is a considerable gap in knowledge about how the sensitization of neurons changes in arthritic pain in large animals, it is clear that DRG neurons can be cultured from large animals and that patch clamp analysis could be conducted. Therefore, the arthritic pain community would benefit if current investigators using large animals in the field establish collaborations with those with patch clamp electrophysiology skill set.

#### 5.3.4 Functional assays: Ca^2+^-imaging

Although electrophysiology is considered to be the gold standard for recording neuronal activity, there are several caveats of the technique as discussed previously. An alternative technique is Ca^2+^-imaging, which is a less technically demanding technique and provides an indirect measurement of cellular response and, in neurons, AP firing by algogens. In addition, Ca^2+^ signals in the nucleus can regulate gene transcription and an increase in intracellular Ca^2+^ can release neurotransmitter that has both short- and long-term effects (Berridge et al., 2003, Lyons and West, 2011). Therefore, quantifying the intracellular [Ca^2+^] in response to different stimuli offers distinct advantages to understanding pain mechanisms. The two major breakthroughs that enabled imaging and quantification of Ca^2+^ signals in cells were the development of fluorescent Ca^2+^ indicators, such as fura-2 and fluo-3, and the development of genetically encoded Ca^2+^ indicators (GECIs), both from the laboratory of Roger Tsien (Miyazawa et al., 1998, Tsien, 1980). The principle underlying fluorescent Ca^2+^ indicators is that these dyes undergo large increases in fluorescence (or spectral shifts) depending upon the amount Ca^2+^ bound and can be either non-ratiometric (excited by one wavelength of light) or ratiometric (can be excited by more than one wavelength of light, e.g. fura-2, or have a dual emissions peak, e.g. indo-1). For example, the commonly used non-ratiometric fluophore for imaging neurons, fluo-4, can be efficiently loaded into cells in salt form or acetoxymethyl ester form, has an absorbance wavelength of 488 nm and has low Ca^2+^ binding affinity thus making it suitable for imaging a broad range of cells using microscopes equipped with standard fluorescein filter sets (Gee et al., 2000). In comparison, a ratiometric Ca^2+^ indicator like fura-2 allows for more precise quantitative measurements and comparison of Ca^2+^ signals because it is excited at 350 and/or 380 nm thus allowing for ratioing of the signals. Specifically, the dye is excited at 380 nm in the Ca^2+^ free form (resting fluorescent signal) and at 350 nm in the Ca^2+^ bound form, both of which emits at 500 nm. Dividing these two emitted fluorescence values gives an accurate measure of Ca^2+^ concentration and cancels out the effects of differential dye loading and photobleaching between experiments (Paredes et al., 2008).

A large number of cells can be imaged at the same time using this technique and it has provided useful insights into pain signaling mechanisms. For example, DRG neurons have been profiled based on their intracellular Ca^2+^ response to a multitude of algogens in order to functionally distinguish between the different neuronal subtypes (Teichert et al., 2012). Furthermore, Ca^2+^ imaging of FLS has revealed the link between an increase in intracellular Ca^2+^ via acid-sensing ion channel 3 (ASIC3) and cell death, a pathway that might be important in understanding arthritic inflammation and pain (Gong et al., 2014).

To enable *in vivo* Ca^2+^ imaging, GECIs have also been developed, with the GCaMP family being the current GECI of choice for neuroscientists (Anderson et al., 2018b). This technique has been used to visualize some fundamental somatosensory pathways, such as identification of unmyelinated sensory fibers expressing the G protein-coupled receptor, MRGPRB4, that detects massage-like stroking of hairy skin (Vrontou et al., 2013). *In vivo* Ca^2+^ imaging has also helped visualize the polymodality of nociceptors and increase in DRG neuron excitability following induction of an inflammatory environment (Chisholm et al., 2018, Emery et al., 2016). However, the proportion of observed polymodal nociceptors differed between the studies of Emery et al and Chisholm et al, possibly due to the different methods utilized to stimulate nociceptors (i.e. order of mechanical and thermal stimuli application), as well as differences in the statistical tools utilized to analyze the data. Application of this technology on large animals could further validate the extent of polymodality of nociceptors innervating the skin and, more importantly for the field of arthritis, joints. Indeed, *in vivo* imaging of knee-innervating DRG neurons in GCaMP3 mice has revealed increased response to noxious mechanical stimuli following DMM compared to the same neurons in healthy mice, thus directly relating pain behavior to neuronal function (Miller et al., 2017). However, the apparatus required for conducting *in vivo* imaging (e.g. anesthesia combined with microscopy) might preclude such analysis in larger animals becoming a standard experimental procedure.

In addition to the practicalities involved, the major disadvantage of Ca^2+^ imaging is that it is an indirect measure of AP firing and a subthreshold increase in intracellular Ca^2+^ can be mediated via ion channels such as, TRP channels, Ca_V_s, NMDA receptors, α-amino-3-hydroxy-5-methyl-4-isoxazolepropionic acid (AMPA) receptors, and/or through Ca^2+^ release from internal stores through inositol 1,4,5-trisphosphate receptors (IP_3_Rs) and ryanodine receptors (reviewed in (Grienberger and Konnerth, 2012, Taylor et al., 1999)). Indeed, a recent analysis demonstrates that GECIs are not suitable for resolving high frequency (>3 Hz) AP firing in cultured trigeminal neurons (Hartung and Gold, 2020). Therefore, efforts have been made to simultaneously perform Ca^2+^ imaging and patch clamp on DRG neurons (Hayar et al., 2008).

Similar to patch clamp electrophysiology, very few studies have investigated large animal neurons using Ca^2+^ imaging in the context of nociception. *In vitro* Ca^2+^ imaging of canine DRG neurons demonstrated their ability to respond to algogens such as, capsaicin and pruritogens such as, histamine (Ganchingco et al., 2019). Similarly a recent *in vitro* study imaged sheep DRG neurons to show hypoxia and acidosis induced increase in Ca^2+^ response (Ma et al., 2020). Ca^2+^ imaging of neurites from porcine DRG neurons has also revealed that “silent” nociceptors (characterized by tetrodotoxin-resistance) are likely to have larger amplitude Ca^2+^ transients upon electrical stimulation (Jonas et al., 2015). These studies provide evidence that functional assays developed in rodents can be adopted in large animals and that they warrant future investigation using these techniques in the field of arthritic pain.

## 6 A recommendation to leverage large animals to understand cellular pain mechanisms

Given that musculoskeletal disorders are the principle contributing factor to the years lived with disability index of the global disease burden (Vos et al., 2012), there is an urgent need to understand mechanisms of arthritic pain and this review has highlighted how large animals can help in this endeavor by providing a more anatomically appropriate alternative to rodents. It is clear from the discussion above that proof-of-concept studies demonstrating the *in vitro* models and techniques described can be adapted to large animal research. We propose that utilizing *in vitro* assays established in the rodent pain field in large animals, to complement the *in vivo* studies already being conducted, can provide answers to major outstanding questions in the arthritic pain field with regard to if and how neuronal properties change during naturally occurring arthritis and how peripheral non-neuronal cells facilitate nociception. Insights gained from studying large animals are likely to be more relevant to clinical translation than those arising from studies with rodents, with the added benefit of being easier to conduct than research with human tissues because animal tissues can be obtained from veterinary research facilities, farms, abattoirs and veterinary biobanks (e.g. the Cornell Veterinary BioBank or Vetmeduni Vienna VetBioBank). However, if more pain studies on large animals are to be conducted, it will require collaboration between veterinary practitioners, clinicians and basic scientists along with co-operation of funding agencies. An analysis of published articles on veterinary sciences showed that research that does not involve zoonotic diseases with animal vectors (e.g. Lyme disease and influenza), is less likely to receive funding, and is more likely to be published in lower impact factor journals, compared to human biomedical research (Ducrot et al., 2011). However, given the potential of large animal research leading to the discovery of breakthrough pain relief in both humans and animals, a concerted effort needs to be made at organizational and personal level in keeping with the philosophy of “one medicine” which recommends cooperation between human and animal health (Zinsstag et al., 2005).

## Author contributions

S.C. wrote the review with assistance from M.A., F.M.D.H and E.St.J.S. All authors approve the final version of the article.

## Declaration of Competing Interest

The authors declare that there is no conflict of interest regarding the publication of this paper.
